# Dielectrolysis

**Published:** 1885-11

**Authors:** 


### ANOTHER WONDERFUL THERAPEUTIC APPLICATION—"DIELECTROLYSIS."

M. BRONDEL has made a communication to the *Paris Academie de Medecine* on the direct medication of the internal organs, by what he terms *dielectrolysis*, meaning thereby "the electrolytic decomposition of a chemical compound, and the forcing of its active medicinal constituents *through* the tissues of the body by virtue of their affinity for one of the poles of a galvanic battery." The *New York Medical Journal*, from which we extract the item, considers this "the most notable therapeutic novelty" that has come up since the anæsthetic power of cocaine was announced.

"M. Brondel, of Alffiers, brought forward a novel plan of medication at a recent meeting of the Paris *Academie de Medecine* ("Rev. med."). By the term "dielectrolysis" (*dielectrolyse*) he refers to a process for making a nascent chemical substance pass through the tissues. For example, taking iodine, a body which is readily "dielectrolyzable," he applies to any desired part of the person a compress wet with a solution of iodide of potassium, and over it he places the negative electrode of a galvanic battery, the positive electrode being held against any indifferent part of the body. The iodine leaves the potassium, and, actually traversing the intervening tissues, rapidly arrives at the positive electrode, as may be ascertained by testing with starch paper. In effect, therefore, this is a hypodermic, or rather interstitial (*intra-organique*), method without wounding the skin or producing pain. As a great number of simple bodies may thus be made to penetrate from one point to another, the practical applications of the new method may be very numerous and very important. By it the author has cured fibrous tumors of the uterus, a case of perimetritis, a rheumatic ovarian neuralgia, and several cases of chronic rheumatism. He has in view further trials upon parasitic and malignant tumors, diseases of the skin, syphilis, neuralgias, etc., and especially pulmonary consumption, on which latter he proposes to try the action of various mineral antiseptics, such as arsenic, mercury, fluorine etc."

[Subsequent events proved this to be a mistake—no such change occurred.—Ed.]

